# Supplementary figures and images for: Identification and expression analysis of the DREB transcription factor family in pineapple (Ananas comosus (L.) Merr.)
Source: PeerJ. 2020 Apr 28;8:e9006. doi: 10.7717/peerj.9006 (PMC7194095; doi:10.7717/peerj.9006)

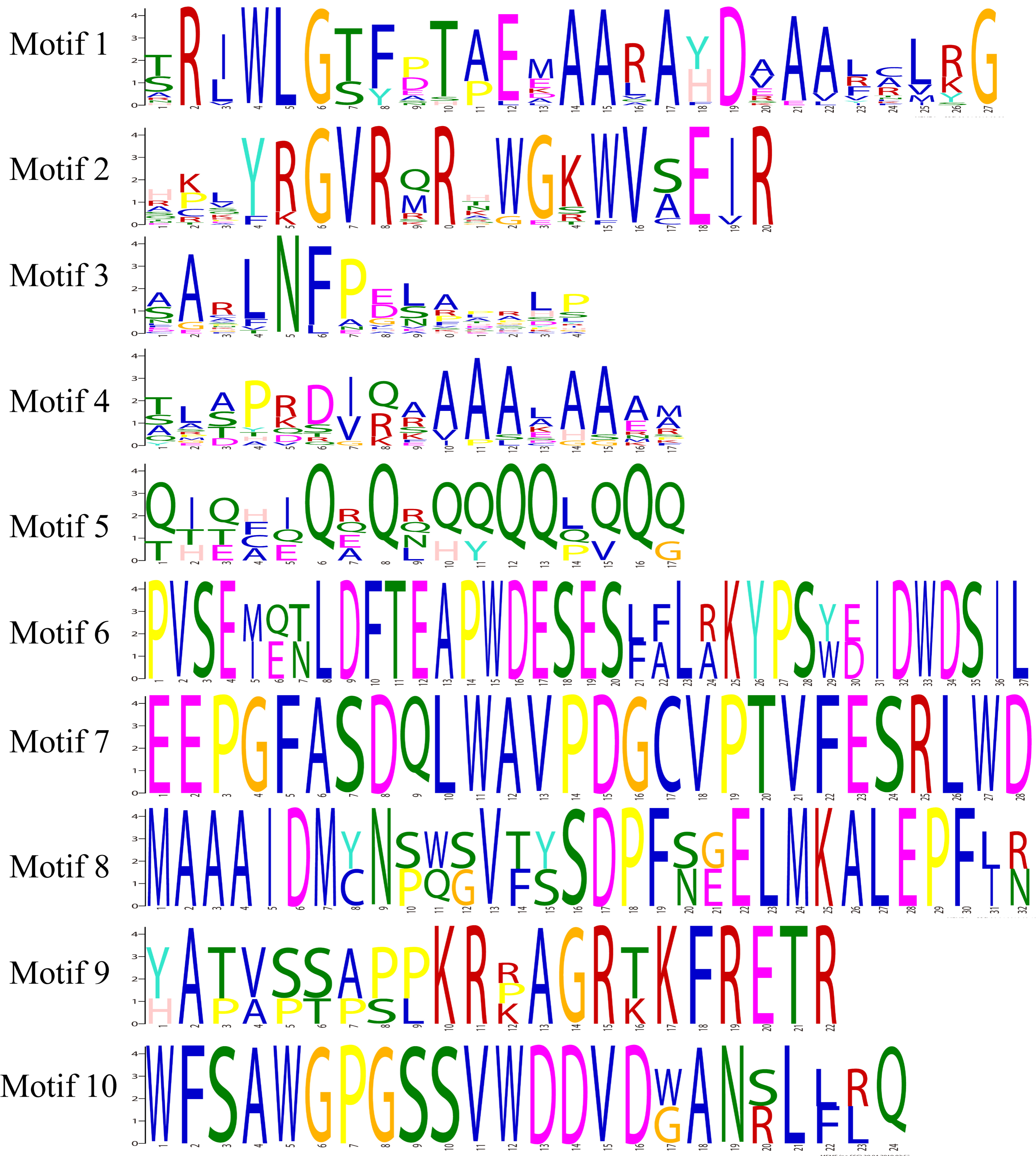

Supplement: Supplemental Information 1 [file peerj-08-9006-s001.pdf]
